# Variante anatómica y acceso venoso: el desafío de la vena cava izquierda persistente en neonatos

**DOI:** 10.47487/apcyccv.v6i1.455

**Published:** 2025-02-12

**Authors:** Arianna Sofia García Calle, Vanessa Patricia Moreno Ccama, Ángel Francisco Samanez Obeso

**Affiliations:** 1 Universidad Científica del Sur, Lima, Perú. Universidad Científica del Sur Universidad Científica del Sur Lima Peru; 2 Instituto Nacional de Salud del Niño, San Borja , Lima, Perú. Instituto Nacional de Salud del Niño, San Borja Lima Perú

*Sr. Editor*

La vena cava superior izquierda persistente (VCSP) es la anomalía venosa torácica más frecuente y forma parte de condiciones cardíacas complejas. Anatómicamente, se origina en la unión de las venas subclavia izquierda y yugular interna, discurre por el lado izquierdo del mediastino, adyacente al arco aórtico, y drena principalmente en la aurícula derecha a través del seno coronario (SC). La prevalencia de la VCSP es del 0,3 - 0,5% en la población general, llegando hasta el 12% en pacientes con cardiopatías congénitas. Esta anomalía coexiste con la vena cava superior derecha en el 80-90% de los casos.

Aunque a menudo es asintomática y se detecta incidentalmente, cuando no está asociada a otras anomalías cardiacas puede causar problemas significativos como arritmias y cianosis ^1^^-^^4^. Esta condición, aunque es infrecuente, tiene implicaciones importantes en la colocación de catéteres de inserción periférica central (PICC), que se utilizan comúnmente en unidades de cuidados intensivos neonatales (UCIN).

Esto ocurre por la incapacidad de la vena cardinal anterior izquierda para regresar a su estado normal durante el desarrollo embrionario. El sistema venoso primitivo está formado por tres pares de venas: vitelinas, umbilicales y cardinales. Las venas cardinales superiores e inferiores son esenciales para el retorno sanguíneo al corazón primitivo desde las secciones craneal y caudal del embrión ^4^. Estas venas se unen para formar las venas cardinales comunes, que drenan en el seno venoso bicorne. La sección caudal de la vena cardinal superior derecha, junto con la vena cardinal común, origina la vena cava superior derecha ^3^^,^^4^ Normalmente, la vena cardinal común izquierda y la porción caudal de la vena cardinal superior izquierda se reabsorben; si esto no ocurre, persisten como la vena cava superior persistente ^4^. Esto provoca que la vena drene hacia el seno coronario en lugar de seguir el recorrido habitual de la vena cava derecha hacia la aurícula derecha ^3^. Por lo tanto, la dilatación del seno coronario puede ser el único hallazgo, resultado del flujo sanguíneo desde la vena cava superior izquierda ^1^.

Por lo general, se considera un descubrimiento asintomático en estudios de imagen diagnóstica, aunque puede ser significativo durante procedimientos como la inserción de un acceso venoso central, la monitorización de la presión arterial pulmonar o la colocación de catéteres de dispositivos ^2^. Por ello, se considera que la identificación de esta variante anatómica puede complicar el acceso venoso central y, por ende, afectar la correcta ubicación de un catéter PICC, aumentando el riesgo de mal posicionamiento y complicaciones como arritmias o perforaciones vasculares.

La falta de reconocimiento previo de esta condición puede resultar en intentos fallidos de canulación y en la necesidad de exámenes adicionales, como ecocardiogramas o fluoroscopias, para confirmar la anatomía venosa. Es crucial conocer la existencia de VCSP de antemano en procedimientos invasivos tales como la inserción de un catéter venoso central (CVC), cables de terapia de resincronización cardíaca o implantación de marcapasos ^4^. En nuestra experiencia en la UCIN, en esta última semana se presentaron dos casos sobre la persistencia de la vena cava izquierda las cuales pudieron evidenciarse mediante una Angio-TEM con contraste. Por lo tanto, debe reconocerse correctamente e informarse explícitamente en los informes radiológicos, incluso cuando se trate de un hallazgo incidental ^4^.

La presencia de VCSP puede complicar el acceso al lado derecho del corazón o a la vasculatura pulmonar al utilizar la vena subclavia izquierda, lo que aumenta el riesgo de posicionamiento incorrecto de dispositivos médicos como líneas centrales o marcapasos ^4^^,^^5^. Para evitar complicaciones, se recomienda realizar el acceso a través de la vena subclavia derecha en estos pacientes ^5^. Es fundamental que los profesionales de la salud tanto médicos, enfermeras y radiólogos involucrados en la atención neonatal estén familiarizados con la VCSP para evitar complicaciones en la colocación de dispositivos invasivos, especialmente en pacientes prematuros y críticamente enfermos.

La significación clínica más importante del diagnóstico prenatal de VCSP es su relación con defectos cardíacos y extracardíacos ^6^. Se informa que la VCSP se acompaña de anomalías cardíacas adicionales en el 80% de los casos, y de anomalías extracardiacas adicionales en el 51,5% de los casos ^7^. Sugerimos que se realicen estudios futuros para explorar la prevalencia de la VCSP en neonatos, con el objetivo de facilitar una detección temprana y mejorar el pronóstico a largo plazo. Asimismo, recomendamos que los estudios próximos aborden las complicaciones asociadas a esta anomalía y las mejores prácticas para la colocación de catéteres PICC en estos pacientes.

En conclusión, aunque la vena cava superior izquierda persistente (VCSP) es una anomalía poco frecuente, su reconocimiento temprano es fundamental para evitar complicaciones durante procedimientos como la colocación de catéteres venosos centrales. La identificación de esta condición puede marcar una diferencia crucial al prevenir mal posicionamiento, arritmias o perforaciones vasculares, particularmente en entornos como la UCIN. Finalmente, considerar esta variante anatómica también debe llevar a la búsqueda proactiva de malformaciones cardíacas y extracardíacas asociadas.

Agradezco la oportunidad de llamar la atención sobre este tema y espero que esta carta fomente una mayor conciencia y discusión en la comunidad médica.

## Fuente de financiamiento:

Autofinanciado
